# Suicidal ideation in older people, a public health matter

**DOI:** 10.1192/j.eurpsy.2023.1786

**Published:** 2023-07-19

**Authors:** R. B. Cohen, I. M. Pereira, M. M. Garrido, B. V. Ferreira, M. C. Sousa

**Affiliations:** 1 Clínica 5; 2 Clínica 4, Centro Hospitalar Psiquiátrico de Lisboa, Lisboa, Portugal

## Abstract

**Introduction:**

Suicide in older people is a critical public and mental health issue which requires attention, given that the ageing population is increasing.

Multiple factors, including biological, psychological, and social stressors increase suicidal susceptibility. Unfortunately, elderly are more susceptible to these, such as psychiatric disturbances, physical comorbidities, prior suicide-related behaviours, lack of social support, grief, and increased difficulty in problem-solving

**Objectives:**

In order to review the risk and protective factors, assessment and prevention of suicide in older adults.

**Methods:**

Bibliographic research through PubMed and Web of Science.

**Results:**

Older people can be subdivided into three age groups (from “young old” at 65 years old to “oldest old” after 85 years of age), with suicide being more prevalent in the oldest-old, and overall in men above 75 years old.

Previous psychiatric background, suicidal attempts, substance abuse, poor physical health or disability, family psychiatric history, low social support or isolation, and finantial stress most frequently predispose to suicidal ideation, suicide attempts or death by suicide in this community.

Besides this, ageing relates to a tendency to cognitive impairment, which affects coping mechanisms, leading to deficits in reasoning and decision-making under stressful circumstances during depressive episodes. This can mediate suicidal ideation and associates to greater lethality methods. Geriatric suicidal attempters have been shown to have greater degrees of cortical and subcortical cerebral areas, including the frontal, parietal and temporal regions, as well as significant loss of volume in the dorsomedial prefrontal cortex, insula, midbrain, cerebellum, lentiform nucleus and putamen. Abnormalities in these regions can impair executive and cognitive function, attention, problem solving and ultimately be responsible for suicidal behaviour.

On the other hand, there are suicide protective elements such as physical and cognitive fitness, quality of life and life satisfaction, marital status, religiousness and social support. A prompt identification of modifiable risk factors and strengthening the protective ones by health professionals can reduce this prospect.

**Conclusions:**

Suicidal ideation in older people is a multifactorial public health concern given the very high frequency of completed suicides in this population. Therefore, it is urgent to review and further research to build more effective suicide prevention strategies.

**Disclosure of Interest:**

None Declared

